# Computational Evaluation of Cochlear Implant Surgery Outcomes Accounting for Uncertainty and Parameter Variability

**DOI:** 10.3389/fphys.2018.00498

**Published:** 2018-05-23

**Authors:** Nerea Mangado, Jordi Pons-Prats, Martí Coma, Pavel Mistrík, Gemma Piella, Mario Ceresa, Miguel Á. González Ballester

**Affiliations:** ^1^BCNMedTech, Universitat Pompeu Fabra, Barcelona, Spain; ^2^International Center for Numerical Methods in Engineering, Barcelona, Spain; ^3^Med-EL, Innsbruck, Austria; ^4^ICREA, Barcelona, Spain

**Keywords:** cochlear implant, surgical outcomes prediction, automatic framework, uncertainty analysis, finite element models, computational modeling, monte carlo, probabilistic collocation method

## Abstract

Cochlear implantation (CI) is a complex surgical procedure that restores hearing in patients with severe deafness. The successful outcome of the implanted device relies on a group of factors, some of them unpredictable or difficult to control. Uncertainties on the electrode array position and the electrical properties of the bone make it difficult to accurately compute the current propagation delivered by the implant and the resulting neural activation. In this context, we use uncertainty quantification methods to explore how these uncertainties propagate through all the stages of CI computational simulations. To this end, we employ an automatic framework, encompassing from the finite element generation of CI models to the assessment of the neural response induced by the implant stimulation. To estimate the confidence intervals of the simulated neural response, we propose two approaches. First, we encode the variability of the cochlear morphology among the population through a statistical shape model. This allows us to generate a population of virtual patients using Monte Carlo sampling and to assign to each of them a set of parameter values according to a statistical distribution. The framework is implemented and parallelized in a High Throughput Computing environment that enables to maximize the available computing resources. Secondly, we perform a patient-specific study to evaluate the computed neural response to seek the optimal post-implantation stimulus levels. Considering a single cochlear morphology, the uncertainty in tissue electrical resistivity and surgical insertion parameters is propagated using the Probabilistic Collocation method, which reduces the number of samples to evaluate. Results show that bone resistivity has the highest influence on CI outcomes. In conjunction with the variability of the cochlear length, worst outcomes are obtained for small cochleae with high resistivity values. However, the effect of the surgical insertion length on the CI outcomes could not be clearly observed, since its impact may be concealed by the other considered parameters. Whereas the Monte Carlo approach implies a high computational cost, Probabilistic Collocation presents a suitable trade-off between precision and computational time. Results suggest that the proposed framework has a great potential to help in both surgical planning decisions and in the audiological setting process.

## 1. Introduction

Computational models have shown the potential to predict the performance of implantable devices, providing valuable information to guide pre-operative decisions, assisting surgical planning and supporting implant optimization processes. Although they are not yet used in the daily clinical practice, they have provided promising results for the prediction of cochlear implantation (CI) outcomes (Kalkman et al., 2014; Ceresa et al., 2015; Malherbe et al., 2015; Nogueira et al., 2016). CI is a surgical procedure that aims at restoring functional hearing via an implanted device that electrically stimulates the auditory nerves. Over the last decades, technological advances have helped to significantly improve speech perception in implanted patients. Yet, some cases show suboptimal results, and we contend that this is partly due to a lack of appropriate surgical planning tools.

Advanced computational modeling and simulations could help to guide and assist pre and post-operative decisions to optimize the surgical outcome. However, computational studies that consider a set of pre-defined parameters may lead to inaccurate results since they do not account for the inherent uncertainty of model parameters, or the large inter-patient variability. This uncertainty and parameter variability have been shown to affect CI outcomes (Finley et al., 2008; van der Marel et al., 2014). Patient-specific cochlear anatomy has been identified as one of the main factors that determine intra-cochlear electrode array (EA) position (van der Marel et al., 2014). However, it presents a large variability across patients, leading to a high variation in the EA intra-cochlear position (Finley et al., 2008; van der Marel et al., 2014; Venail et al., 2015) and a broad range of post-operative speech perception scores (Yukawa et al., 2004). Low scores may be the consequence of confused pitch perception or loss of some frequency range due to a mismatch of the alignment between the electrode location and the frequency distribution of the adjacent auditory nerve fibers (ANF) (Rebscher et al., 2008). This causes a harder CI adaptation of the patient, and consequently, a reduction of the possible implant benefits (Rebscher et al., 2008; van der Marel et al., 2014).

Geometrical aspects, such as surgical insertion depth, are not the only factors affecting the CI success. Both geometry and electrical properties of the tissues determine the voltage spread throughout the inner ear. A change in these parameters alters the potential distribution, which is critical to evoke the desired neural response. Tissue electrical resistivity values employed in computational CI models were originally obtained from animal data, and they are still used nowadays (Hanekom and Hanekom, 2016). Nonetheless, electrical properties of bone tissue exhibit the largest variability in humans (Hanekom and Hanekom, 2016). Specifically, bone electrical resistivity has shown to be easily modified by changes of density, which is affected by the chemical composition or some diseases, such as osteosclerosis (Mens et al., 1999). Although the electrical resistivity of the bone has been adapted to a more precise value according to recent studies (Mens et al., 1999; Rattay et al., 2001a; Malherbe et al., 2015), its value cannot be obtained accurately in patients. Hence, the effect of bone tissue on neural excitation profiles remains uncertain.

Despite the large number of techniques employed to study parameter variability and uncertainties in finite element (FE) models (Mangado et al., 2016b), Monte Carlo (MC) method is the most popular because it easily allows generating a set of models – computing for each of them a FE analysis. However, in some studies the associated computational cost is unfeasible when a large set of samples is evaluated, and thus, methods less expensive in terms of computational time are required. In this work, we propose to reduce the computational cost of our study using the Probabilistic Collocation method (PCM), which without modifying the numerical formulation of the FE model, allows evaluating the system outcomes with a reduced number of samples.

Our aim is to study the outcomes of CI computational models considering parameter uncertainty and variability for the prediction of neural response to support optimization processes for surgical planning and implant design. To this end, we make use of our framework for the complete functional assessment of CI (Mangado et al., 2016a), and we combine it with uncertainty quantification methods. First, we study the CI outcomes in a virtual population using the MC method. Due to the high amount of time required for such uncertainty quantification study, a High Throughput Computing (HTC) environment is used to considerably reduce the overall time of computational analysis. Second, we focus on the implant performance in a patient-specific case using PCM. This reduction of the time required for the study allows us to seek the optimal stimulus levels delivered by the implanted electrode – a highly time-consuming process–, providing thus the favorable set up for the implant programming in the given patient during the post-intervention procedure.

## 2. Materials and methods

In this section, first a brief description of the computational framework employed for the evaluation of CI models is introduced (section 2.1). The automatic framework consists of three main blocks: (1) the generation of the computational models, (2) their functional assessment and (3) the evaluation of their outcome. Then, the identification and characterization of the different sources of uncertainty and variability are presented (section 2.2). Finally, uncertainty quantification methods to propagate parameter variability and uncertainty through the CI simulations to the system output are described (section 2.3).

### 2.1. Computational framework for CI assessment

#### 2.1.1. CI computational model generation

**Figure 1:**
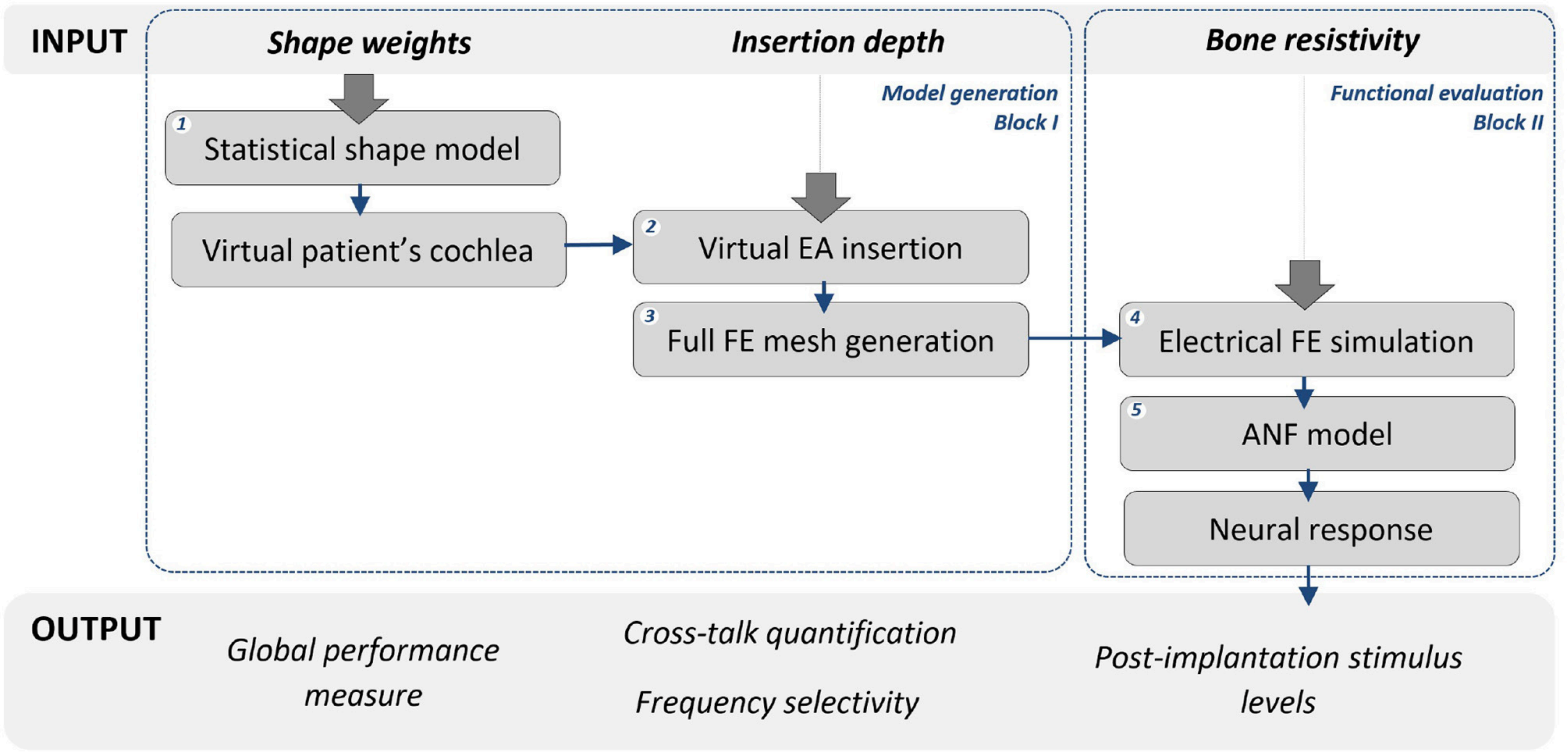
CI evaluation framework. Input variables, for which uncertainty and variability are assessed, are shown at the top level. Their respective arrows indicate the step in which the uncertainty is introduced. Blue arrows show the workflow path of the framework for the two main blocks: model generation and functional evaluation. The evaluated output variables are shown at the bottom level.

**Figure 2:**
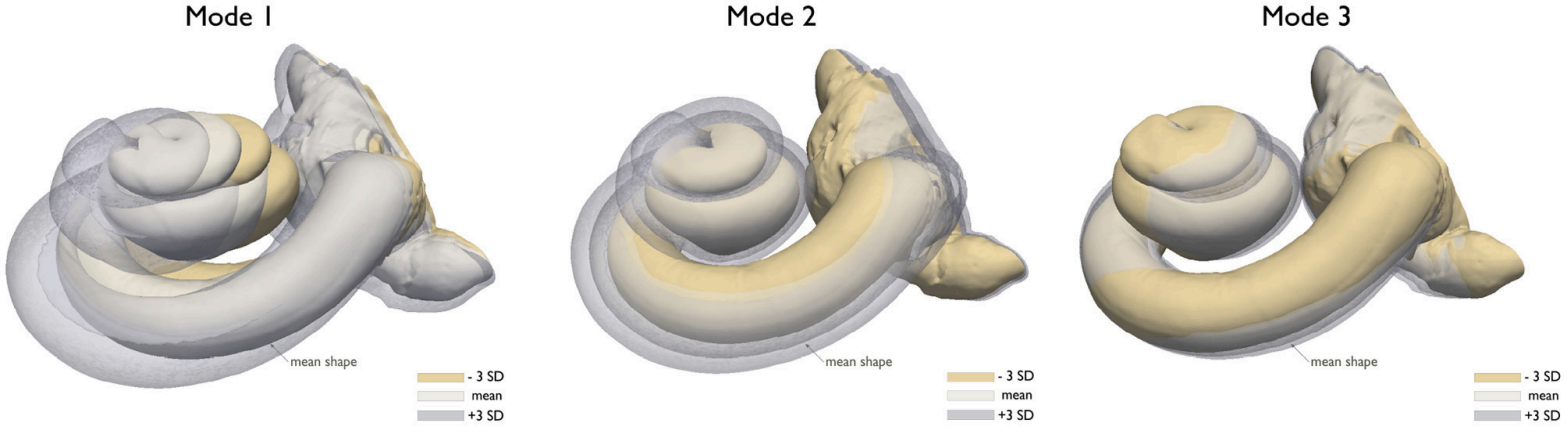
Illustration of the changes of the cochlear morphology by varying the three first modes of variation of the SSM, mean shape, and ± 3 standard deviation (SD) from the mean.

**Figure 3:**
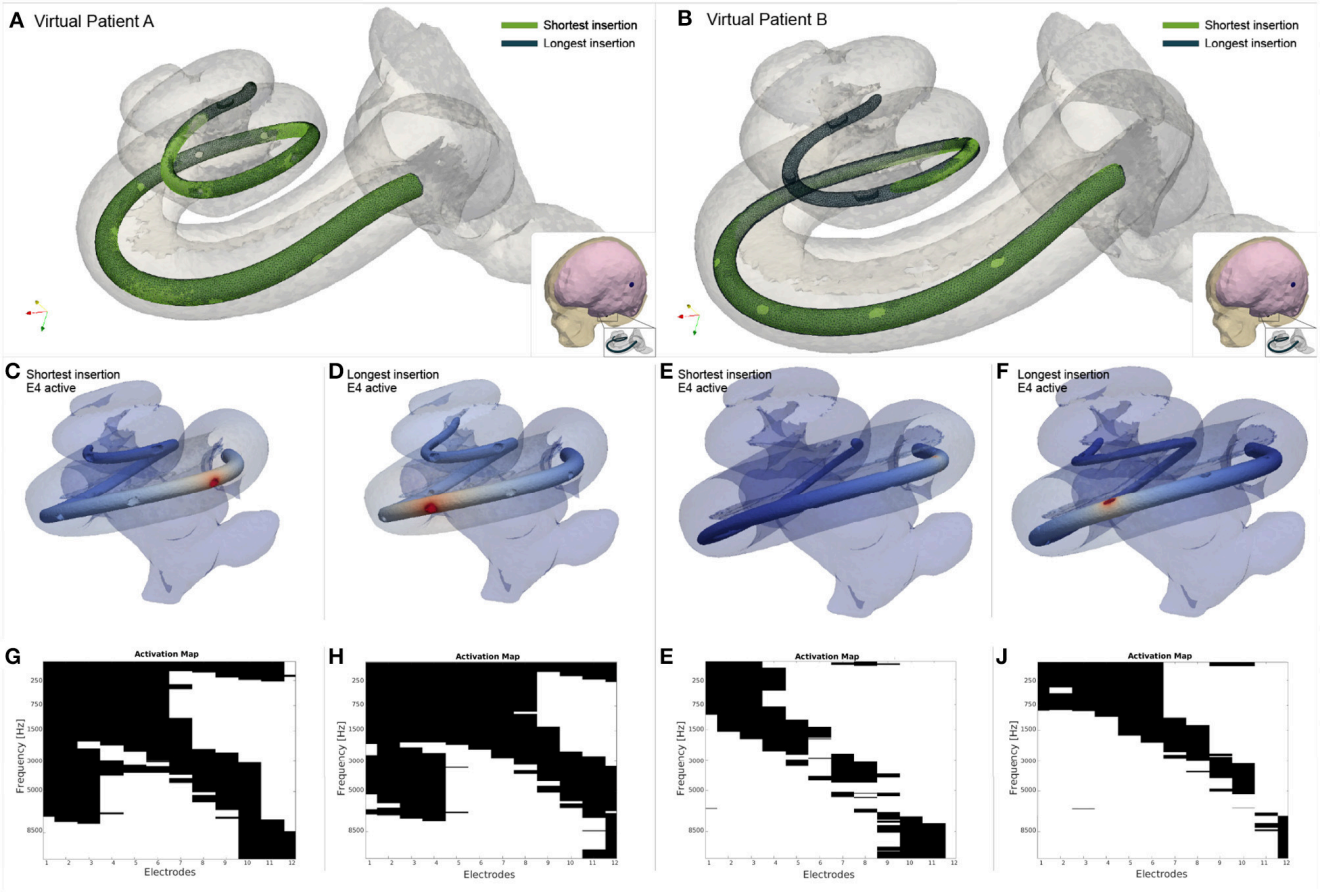
Example of two virtual patients with different cochlear sizes, both with the shortest and longest EA insertion depth allowed by the model morphology. **(A,B)** 3D model of the cochlea. **(C–F)** Potential created by the fourth electrode (E4) on the EA. **(G–J)** Evoked neural response on all ANF when each electrode delivers the stimulus.

The first block of the framework is composed of a statistical shape model (SSM), a virtual insertion algorithm and a three dimensional full model of the head. The SSM is a compact representation learned from a training population of the shapes extracted from imaging data. It encodes the shape variability in the population by a small set of weights modulating the contribution of the main modes of variation around the mean shape (Cootes and Taylor, 1995) (Figure 1 Step 1). By modulating these weights within a limited range, the mean shape of the cochlea is deformed so that anatomically plausible cochlear morphologies are obtained (further implementation details shown by Mangado et al., 2016a; Gerber et al., 2017). Therefore, we can obtain a set of cochlear surfaces, each of them created from a different combination of the scalar weights (Figure 2). Here, this set of surfaces is referred to as population of virtual patients. The surgical trajectory of the EA insertion is computed via our surgical planning software based on the open source simulation framework SOFA (Allard et al., 2007). This surgical trajectory is matched to the centerline of the EA mesh by using a parallel transport frame algorithm (Mangado et al., 2016a). It allows adapting geometrically the EA mesh to the obtained insertion trajectory for a given virtual patient (Figure 1 Step 2). The parametrization of the virtual EA insertion allows having control over the insertion depth (Mangado et al., 2016a). Cochlear anatomies of two virtual patients with two different insertion depths are shown in Figures 3A,B. The EA is based on Med-EL Flex28 design, with 12 electrodes numbered from 1 to 12 as E1 to E12. The virtual patient's cochlea and the array virtually inserted are coupled with a generalized model of the brain, scalp and skull. To further conduct the computational FE simulations, all the elements are transformed into a single volumetric mesh of approximately 2 · 10^6^ tetrahedral elements free of intersections. (Figure 1 Step 3) (Mangado et al., 2017a).

#### 2.1.2. CI functional assessment

The second block encompasses the simulations of the electrical field and the ANF model for the assessment of the evoked neural response. The potential distribution is computed by the FE method (Figure 1 Step 4) considering a monopolar configuration according to the stimulation strategy used by the implant design: one intra-cochlear electrode is set as active source, while the return is defined as the reference electrode located on the scalp (Mangado et al., 2017a). In the current work, the intra-cochlear electrode delivers a biphasic cathodic-first pulse of 100 μs, similar to previous reported studies (Rattay et al., 2001a,b), with an intensity of 350 μA.

The neural response provoked by the activation of the intra-cochlear electrodes is computed by the ANF model (Figure 1 Step 5). This multi-compartment fiber model reproduces the active behavior of the neural cell membrane according to ionic channel kinetics (Hodgkin and Huxley, 1952), adjusted to the human temperature to fit the temporal behavior of the human ANF (Rattay et al., 2001a, 2013). The neural activity is considered as a single spike induced by the depolarization of the neuron, which generates an action potential that is propagated through the ANF. The external stimulation used to initiate this neural response corresponds to the potential value obtained by the FE simulation at the specific spatial location (Rattay et al., 2001a,b). These locations are equal to the ANF compartment coordinates, modeled according to the 3D model of the patient's cochlea and considering the human ANF morphology (Mangado et al., 2016a, 2017a). The model includes 334 nerve fiber bundles. As the human cochlea has approximately 30,000 nerve fibers, each fiber bundle represents 90 neural fibers, retaining enough frequency resolution. Figures 3G–J shows examples of four different neural responses for the presented examples.

#### 2.1.3. CI outcome evaluation

**Figure 4:**
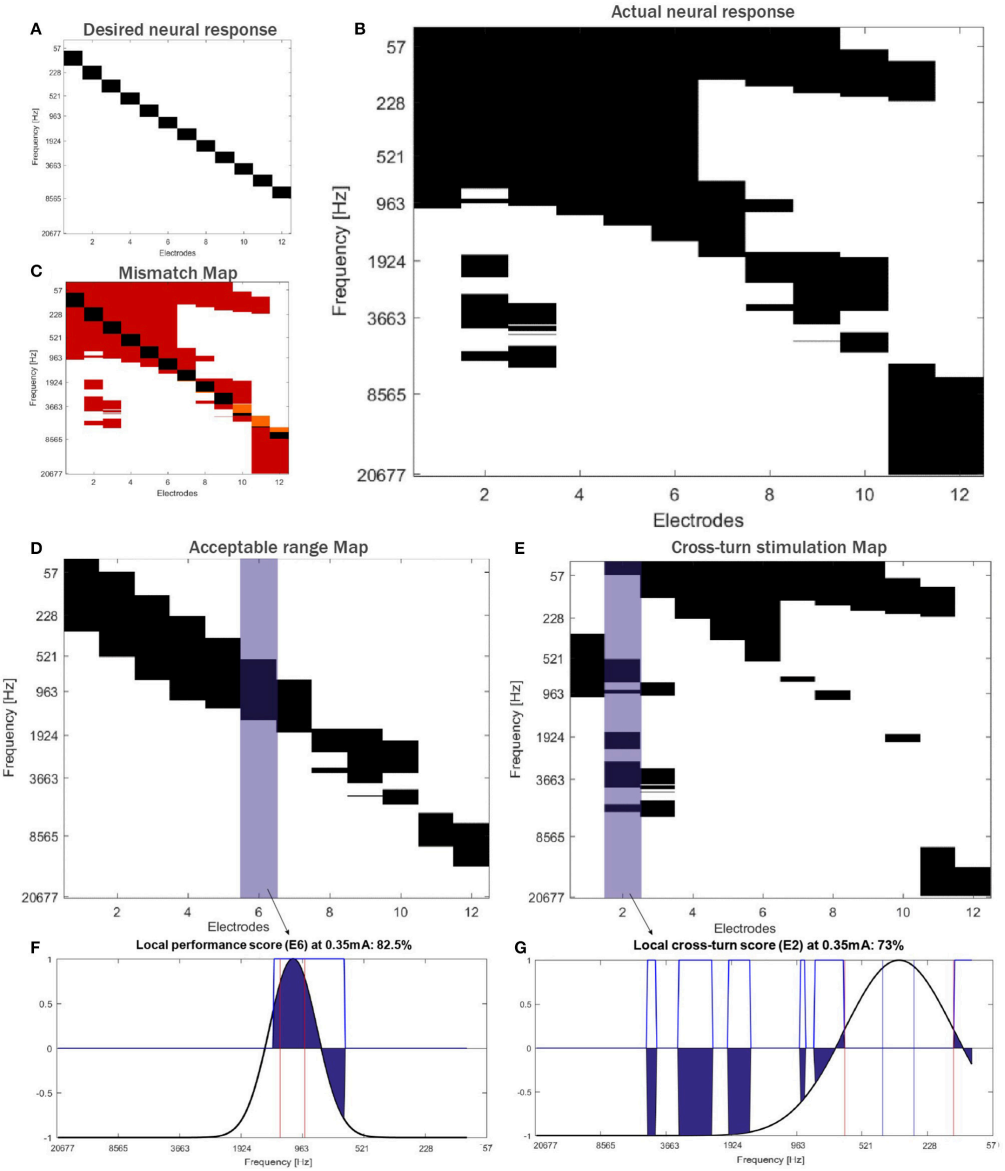
Activation maps for **(A)** the desired and **(B)** the actual neural response, and **(C)** mismatch map computed in a randomly generated virtual patient. Each electrode on the array is numbered, from the tip (E1) to the base of the array (E12). The actual activation map is split and evaluated according to the stimulation found in the half turn of the cochlea where the mid target frequency is located at the middle of the cochlea section evaluated **(D)**. The activation at the rest of the cochlea **(E)** is considered as cross-turn stimulation. Local performance score for E6 **(F)** and local cross-turn score for E2 **(G)**. Activation profiles of both electrodes are highlighted in blue in their corresponding maps.

The third block of the framework assesses the implant performance. Here, the patient's neural response is evaluated by an activation map (Mangado et al., 2017a), where rows represent the frequency bandwidth of each ANF bundle and columns the electrode delivering the stimulus (see Figure 4). A target activation map (Figure 4A) describes the ideal excitation according to the tonotopic map of the cochlea, selectively stimulating the desired ANF. This tonotopic map provides a specific pitch perception according to the location of the evoked ANF–capturing high frequencies at the base and low frequencies at the apex of the cochlea (Greenwood, 1990; Stakhovskaya et al., 2007).

The actual activation map computed by the computational framework (Figure 4B) is then compared with this target map, which leads to a mismatch map (Figure 4C). We propose a set of measures using this mismatch map to quantify the neural response to assess the final CI outcome of the patient. We evaluate the global implant performance by the neural activation specificity –true negative rate. We also evaluate two local effects: the frequency selectivity and the cross-turn stimulation (Figures 4D,E). The frequency selectivity defines the mismatch between excited frequencies due to a non-focused current stimulation. We refer to this measure as the local performance score. Cross-turn stimulation corresponds to the excitation of the ANF that are located half turn further from the desired frequency bandwidth. Therefore, the second local measure, named cross-turn stimulation score, evaluates the non-selective ANF activation (Figures 4F,G).

To compute these two scores, the activation map is split into two–one analyzing the half turn of the cochlea where the center corresponds to the mid target frequency, and another representing the activation at the rest of the cochlea (i.e., cross-turn stimulation) (see Figures 4D,E, respectively). We consider that the target bandwidth of each electrode has a modified Gaussian distribution and, given an activation map, assigns positive and negative values to acceptable (up to 3 mm of bandwidth) and non-acceptable activation, respectively (see Figure 4F). A frequency bandwidth broader than 3 mm would imply a change in tone and a confusing pitch for the patient (Mistrík and Jolly, 2016). Therefore, cross-turn stimulation areas are penalized. This leads to a performance measure, one for each electrode, where the mid value corresponds to a zero stimulation, the maximum to the ideal activation profile and the minimum to the inverse profile, i.e., the activation of all non-desired ANF exclusively. The described performance measure is applied to both maps obtaining for each virtual patient a value of local performance and cross-turn stimulation score for each electrode (Figures 4F,G). For interpretation, both scores are mapped between (0, 100)% (for further details, see Mangado et al., 2017a).

Post-implantation stimulus comprises the stimulation threshold, T-level, and the maximum amplitude of stimulation, C-level. T-level defines the amplitude at which the first neural response within the desired target bandwidth is obtained. The desired target bandwidth is defined according to the EA design. C-level is here considered to be reached when the maximum recruitment of ANF within the desired target bandwidth is accomplished, while minimizing the cross-turn stimulation and avoiding frequency overlap. Therefore, C-level corresponds to the stimulation level of each electrode that provides the highest values of both specificity and sensitivity of the mismatch map.

### 2.2. Uncertainty and variability characterization

Uncertainty and variability sources considered in the current study were the insertion depth of the EA, the cochlear anatomy and the bone electrical resistivity. The EA insertion depth was characterized by a normal distribution with mean μ = 27 mm and standard deviation σ = 1 mm to cover the possible range found in the population. This mean value was reported previously in our computational model—with this cochlear anatomy—to be the most reliable to obtain the best CI outcome, and therefore, considered as the target depth (Mangado et al., 2017b). For the patient-specific study, we considered a standard deviation of 0.5 mm related to the inherent uncertainty due to the surgical insertion procedure.

Since the active stimulation range of the EA design is 23.1 mm, the minimum insertion depth was defined as 24.1 mm (active stimulation range plus 1 mm of the tip of the EA) to ensure a full insertion –all electrode contacts of the EA inside the cochlea. The insertion depth was measured from the round window. We took the deepest insertion allowed by the cochlear duct in cases of large values of insertion depth in cochlear anatomies with small dimensions. Figure 3 shows an example of a small (Virtual patient A) and large cochlea (Virtual patient B)—with 5.5 mm of difference between their Organ of Corti length—with their shortest and longest possible insertions.

We characterized the variability of the cochlear anatomy by modifying the weights of the first three principal components of the SSM (see section 2.1.1). These weights were sampled from normal distributions with mean and standard deviation of 0 and 1, respectively, with maximum values of ±3. This avoids obtaining unrealistic shapes with high deformations, while ensuring plausibility of the shape anatomy. For higher standard deviation values, the generated cochlea presents a larger deformation (see Figure 2). The size of the cochlea was described by the length of the osseous spiral lamina, an inner structure located between the Organ of Corti (around 33 mm) and the modiolus wall (around 15 mm) (Stakhovskaya et al., 2007; Rask-Andersen et al., 2012; Venail et al., 2015), visible on our model and μCT images (Rask-Andersen et al., 2012; Martin et al., 2016). In the patient-specific study, the morphology was considered a known factor, defined as the mean shape of the SSM, with a length of the osseous spiral lamina of 25.3 mm.

Based on recent studies reporting the influence of bone resistivity in CI models (Malherbe et al., 2016), we defined the bone resistivity parameter as normally distributed, with values μ = 65.0 Ω· m and σ = 21.6 Ω· m. These values were obtained matching electric field profiles to clinical data in a small number of computational models considering a broad range of bone resistivity values (Nelson et al., 2008; Tang et al., 2012; Malherbe et al., 2016).

### 2.3. Uncertainty and variability propagation and quantification

We considered two different non-intrusive approaches, which did not modify the described CI framework. The first study used MC sampling to generate a population of virtual patients according to the variability of the cochlear anatomy and the uncertainty sources described in section 2.2. The second study used both MC sampling and PCM to evaluate the neural response in a patient-specific case.

The analysis via MC was performed by a set of individual evaluations that did not depend on each other, so it is easily parallelizable. This allowed us to use a HTC environment called HTCondor, which enables to easily create a grid of computers, maximizing the amount of available computing resources (Thain et al., 2005). MC sampling was implemented in a HTCondor (8 nodes and 40 cores), in both Windows and Linux platforms, to evaluate a large set of patients using our automatic framework (section 2.1). Nonetheless, the MC sampling technique still required to deal with a large number of simulations—leading to a high computational cost—to obtain a satisfactory accuracy. For this reason, to drastically reduce the number of samples, the second study explored the use of PCM to assess the neural response in a patient-specific case, while accounting for the uncertainty sources.

PCM (Loeven and Bijl, 2008) is a numerical technique to solve stochastic differential equations using (Lagrange) polynomial interpolation and Gaussian quadrature. We used PCM to approximate our model's response—treated as a random field—as a weighted sum of *N*_*p*_ Lagrange polynomial functions of the uncertain input parameters. Let *f*(**x**, ω) be a the random field, a function of (deterministic) **x** and the random variable ω, expanded as:

(1) $$\begin{array}{l} f\left(\text{x},\omega \right)\approx \overset{N_{p}}{\underset{i=1}{\sum }}f_{i}\left(\text{x}\right)\cdot L_{i}\left(\xi \left(\omega \right)\right) \end{array}$$

where *f*_*i*_(**x**) is the value of *f*(**x**, ω) evaluated at the interpolation point ω_*i*_—called collocation point—, ξ is the random basis (chosen so that the uncertain input parameter is a linear transformation of ξ) and *L*_*i*_ the Lagrange interpolating polynomial chaos of order *n* = *N*_*p*_−1 corresponding to ω_*i*_ (i.e., *L*_*i*_(ξ(ω)) passes through the *N*_*p*_ collocation points, with *L*_*i*_(ξ(ω_*j*_) = δ_*ij*_)) (Loeven et al., 2007).

The statistics (mean and variance) are obtained by a Galerkin projection on the polynomial basis, with the collocation points calculated as the points of the Gaussian quadrature (i.e., for each uncertain parameter, the *N*_*p*_ collocation points correspond to the *N*_*p*_ roots of the polynomial basis) (Webster et al., 1996; Loeven and Bijl, 2008). When multiple uncertain parameters are considered, the collocation points are obtained from tensor products of one dimensional points and a total of (*n*+1)^*p*^ runs (rather than *n*+1) are needed, where *n* is the order of the approximation and *p* the number of uncertain parameters. The mean and variance in the case of two stochastic variables are approximated as:

(2) $$\begin{array}{l} \mu =\overset{N_{p}}{\underset{i=1}{\sum }}\overset{N_{p}}{\underset{j=1}{\sum }}f_{ij}\left(\text{x}\right)\cdot k_{i}\cdot k_{j} \end{array}$$

(3) $$\begin{array}{l} \sigma^{2}=\overset{N_{p}}{\underset{i=1}{\sum }}\overset{N_{p}}{\underset{j=1}{\sum }}{\left(f_{ij}\left(\text{x}\right)-\mu \right)}^{2}\cdot k_{i}\cdot k_{j}, \end{array}$$

where *k*_*i*_ and *k*_*j*_ are the weights of the corresponding collocations points ω_*i*_ and ω_*j*_ that compound the random event ω, being *f*_*ij*_(**x**) the solution of *f*(**x**, ω) evaluated at ω_*i*_ and ω_*j*_. Here, we considered a second order polynomial for the Gaussian quadrature and, therefore, three collocation points (*n*+1) for each random variable were required. Two sources of uncertainty were defined, and thus, ${N_{p}}^{2}=9$ model runs were computed. The same uncertainty characterization was employed using MC sampling to create a set of 250 samples and evaluate the accuracy obtained with PCM.

## 3. Results

### 3.1. Virtual population study

Preliminary results obtained from a population of 300 virtual patients showed a high impact of the bone resistivity variability, which hindered the impact of the variability and uncertainty of other parameters on the patient's neural response. Very low global performance values were related to the activation of (1) all ANF due to the vast spread of excitation or (2) very few ANF due to a highly focused potential distribution. No relevant effects were found regarding the rest of uncertainty and variability sources. These widespread CI outcomes are likely due to the wide range of variability in bone resistivity (Kalkman et al., 2015; Malherbe et al., 2016).

We created thus a second population of 1,000 virtual patients, divided in three groups. Each of them considered the bone resistivity as a fixed input parameter. The first group (Group 1) comprised 500 virtual patients with a bone resistivity equal to the mean value 65.0 Ω· m (section 2.2). The two other groups, with 250 virtual patients each, had a resistivity of − σ (Group 2) and + σ (Group 3) from the mean, with σ = 4.5· m according to previous reported values (Mens et al., 1999; Rattay et al., 2001a; Frijns et al., 2009; Kalkman et al., 2014; Malherbe et al., 2015). We also used this mean and standard deviation to characterize bone resistivity uncertainty in the patient-specific study (section 3.2).

**Figure 5:**
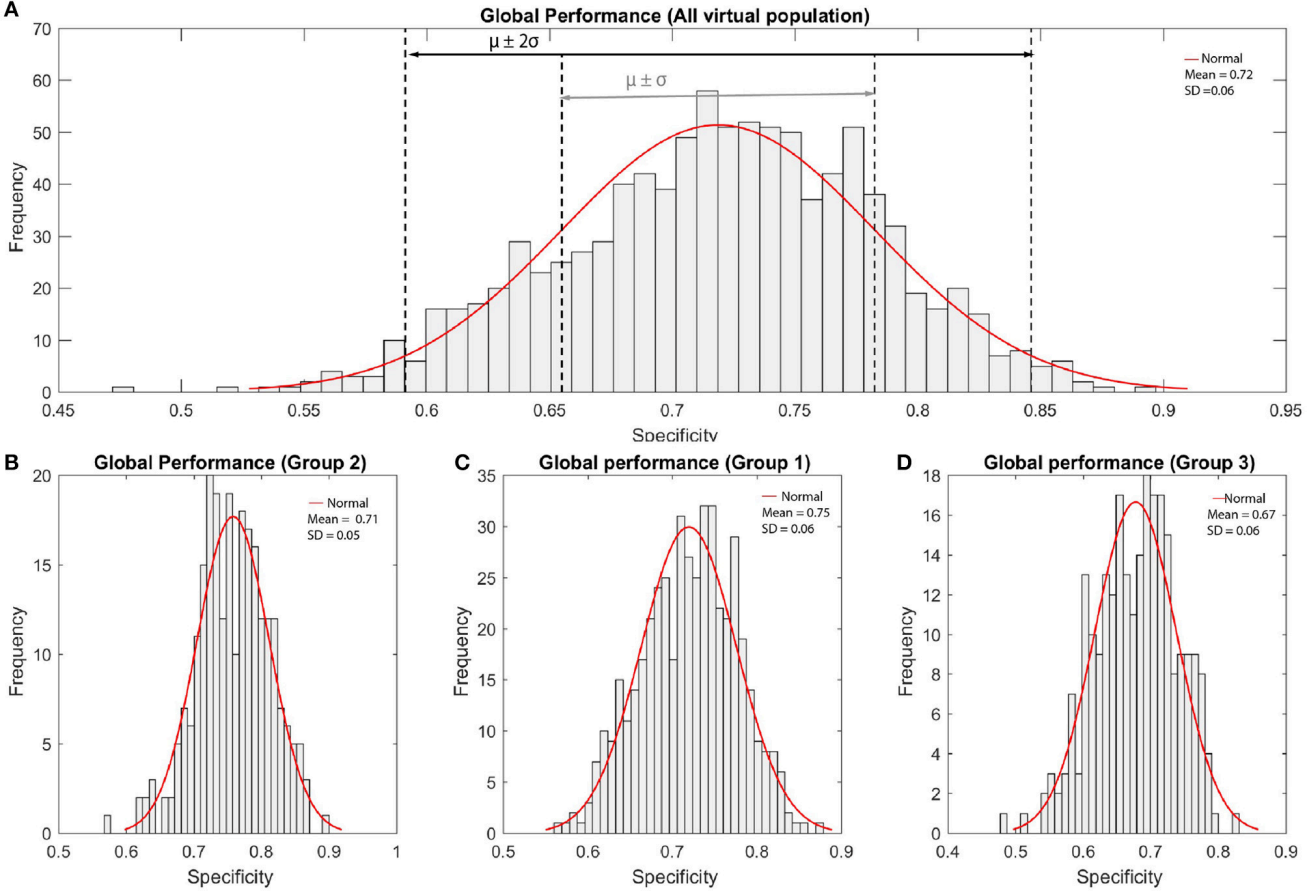
Histograms of global performance of a population of virtual patients. **(A)** All virtual population, **(B)** Group 2 (−1 standard deviation) **(C)** Group 1 (mean value) and **(D)** Group 3 (+1 standard deviation).

The population of virtual patients had an average length of 25.3 ± 1.1 mm and the final insertion depths were 26.7 ± 0.8, 26.9 ± 0.8, and 26.9 ± 0.9 mm for the Group 1, 2, and 3, respectively. Figure 5 shows the CI outcomes for the three virtual populations of patients, with a global performance score (specificity) of 0.75 ± 0.06 (Group 1), 0.71 ± 0.05 (Group 2), and 0.67 ± 0.06 (Group 3).

**Figure 6:**
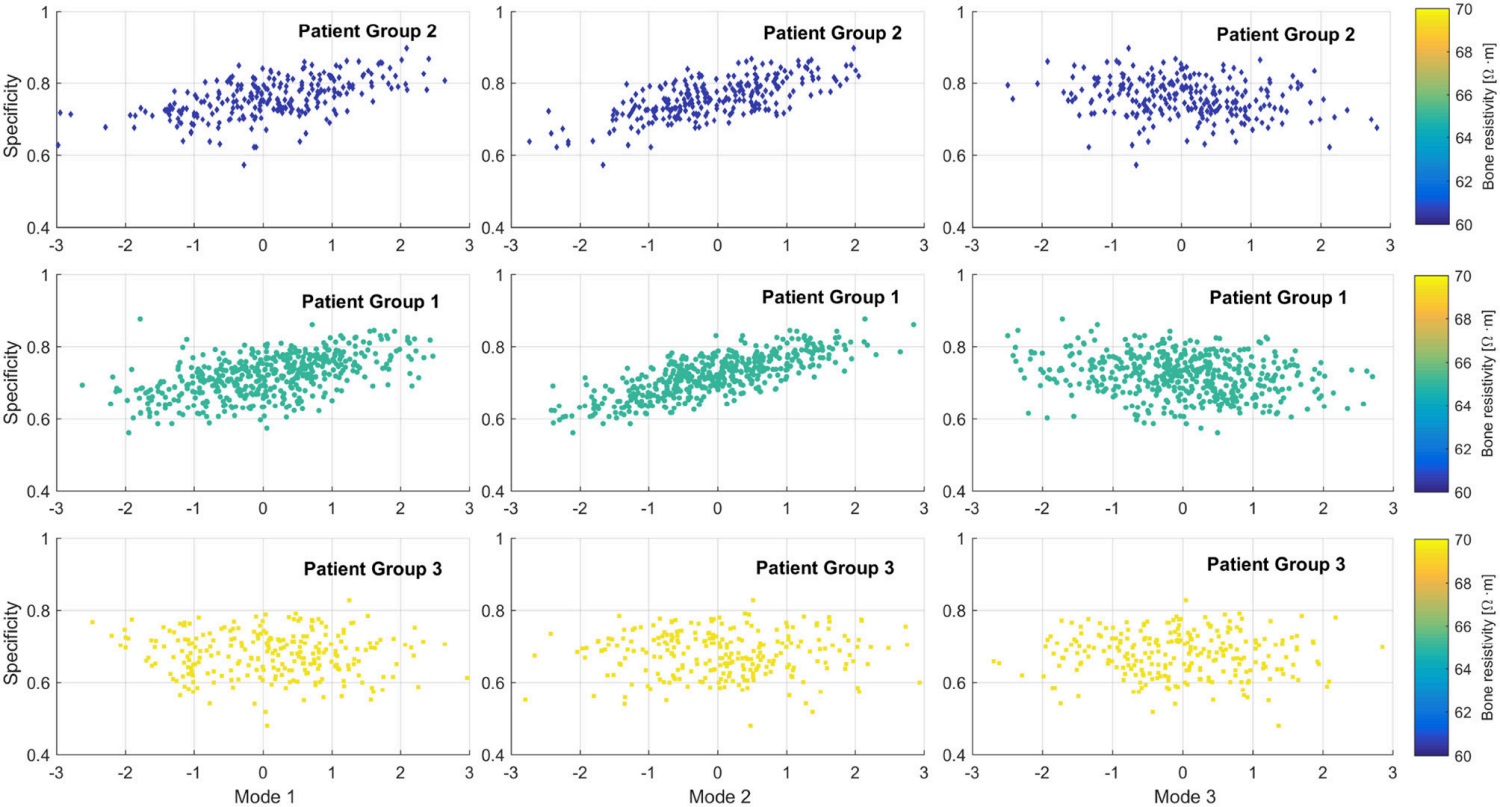
Effect of the cochlear morphology on implant global performance of a population of virtual patients. From left to right, first to third mode of variation. From top to bottom, from low to high bone resistivity values.

Figure 6 represents the global performance according to the shape variability of all virtual patients. The graphics show a clear effect of the bone resistivity on the outcome. In general, lower bone resistivity values led to better global performance measures. Group 3 presented no clear variation related to the morphology. Although the impact of each mode of variation individually was not evident, global performance slightly increased as the second mode took values above the mean. Better results were obtained when the value of the first mode was above 1 standard deviation from the mean, and the third mode, below the mean.

**Figure 7:**
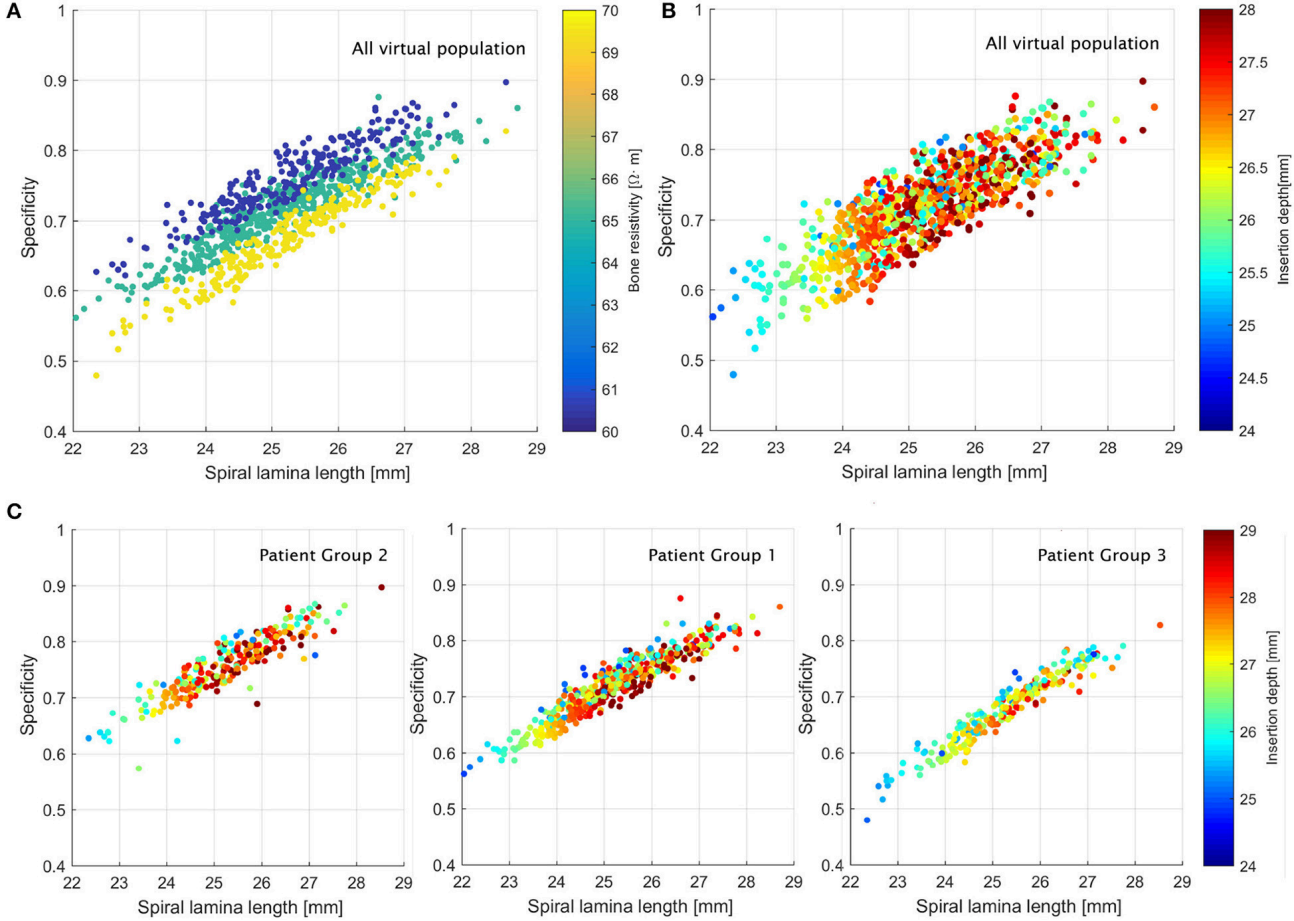
Relation between the global performance and the length of the cochlea **(A,B)** in all the virtual population and **(C)** in each group of patients.

The relation between the global performance and the cochlear length was almost linear: the longer the cochlea, the higher the performance (see Figure 7). The effect of the bone resistivity can also be seen; results improved for longest cochleae with low resistivity values (Figure 7A). Although the insertion depth did not seem to have as large impact as the bone resistivity, some groups with similar behavior were identified (see Figure 7B). Short cochleae with short insertion depth showed the worst results (Figure 7C). Although deepest insertions did not provide the best results in all anatomies, the best outcomes—with global performance score above 0.8—were obtained for insertions deeper than 26 mm in cochleae with a length of the spiral lamina larger than 26.5 mm.

**Figure 8:**
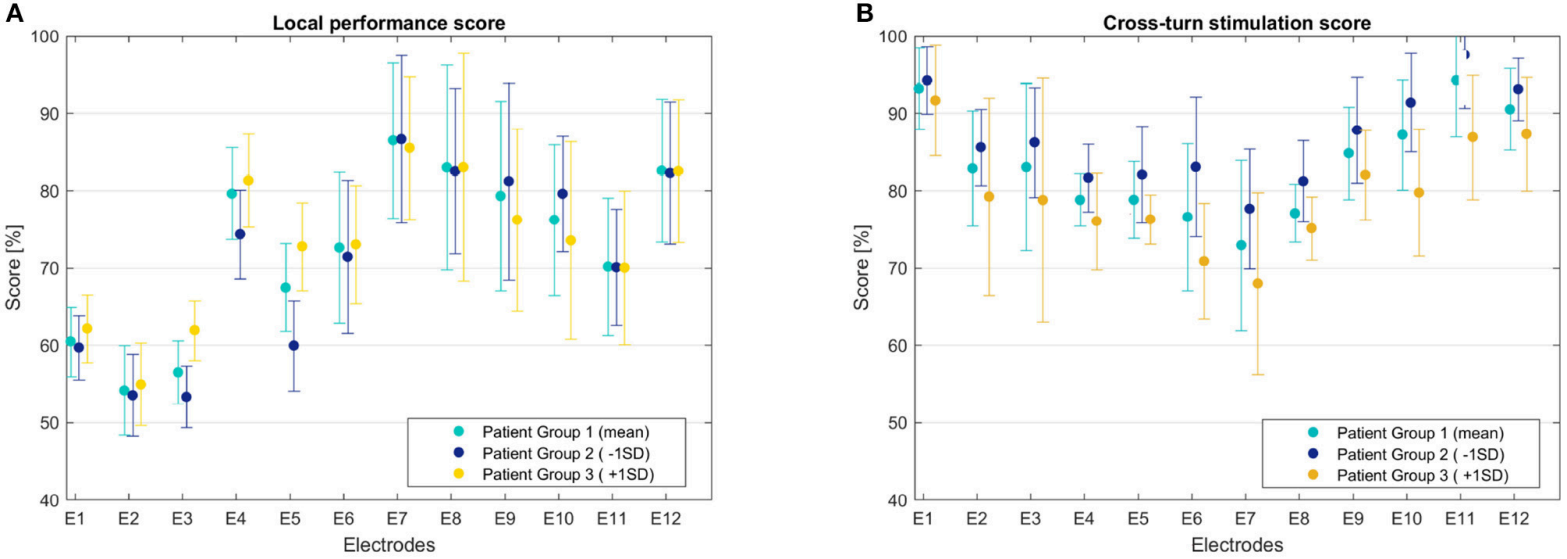
CI outcomes in a population of virtual patients. **(A)** Local performance score, **(B)** Cross-turn stimulation score.

Figure 8 presents the neural response of the three sets of populations of virtual patients with regard to local effects. Apical electrodes performed worse than basal ones, in terms of higher non-focal and non-selective activation, with higher spread of excitation and cross-turn stimulation (Figure 8A). Medial electrodes showed similar cross-turn scores than apical ones, while they presented better local performance scores – more focused ANF recruitment. 34% of all electrodes presented a local performance score higher than 80%, # while less than 9% of all cases obtained a score below 50% and none less of 45%. Cross-turn stimulation scores were 80% of the cases within [70, 95%]. Some outliers (2%) presented the lowest scores below 60 and 13% obtained scores above 95%.

On average, Group 3 obtained the worst performance values due to the higher non-desired ANF excitation and broader spread. Group 2 presented better results in terms of cross-turn stimulation and slightly better in local performance than Group 1. However, for the apical electrodes, Group 2 presented worse local performance score due to the high non-focused activation and missed target frequencies. Group 2 showed slightly narrowed bandwidth, but less non-focused activation, obtaining an overall better performance.

The impact of the insertion depth was also evaluated in terms of local effects. Insertions deeper than 27 mm obtained the best results for apical electrodes (highest values above 90% in E1–E4), although they did not provide such good outcomes in the basal part, missing some target frequencies due to the misaligned electrodes. Group 1 did not show a relevant relationship between the insertion and the local performance. Likewise, cross-turn stimulation was not clearly influenced by the insertion depth, although some of the better results corresponded to insertions between 27 and 28 mm. Some outliers – lowest scores – were identified to correspond to the smallest cochleae (below 24 mm), where the short distances between turns provided a large amount of evoked ANF at non-desired locations. Results of local effects according to the length of the spiral lamina provided similar information, as shown in Figure 7; the smaller the cochlea, the worse the results.

Regarding the computational cost, each patient took 5.1 ± 1.2 h. However, using the HTC environment allowed parallelizing the simulations so that the whole population took <1,010 h (i.e., effective average of 1 h per patient).

### 3.2. Patient-specific case study

**Figure 9:**
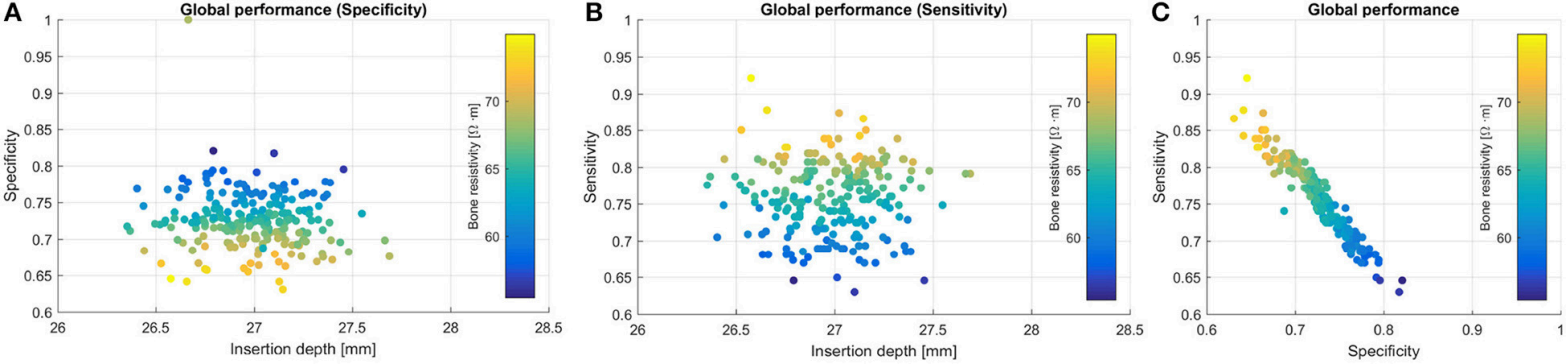
CI global performance of a patient-specific case in terms of **(A)** specificity, **(B)** sensitivity, and **(C)** global performance (specificity vs. sensitivity).

Figures 9A,B shows the global behavior of the patient's neural response using the MC approach. In line with the results presented above, as the bone resistivity decreases, the spread of excitation is narrowed. This causes more focused activation and avoids non-desired stimulation (high specificity values). However, if the spread is too narrow, it may not be able to activate the desired bandwidth (low sensitivity values– see Figure 9C). Bone electrical resistivity has a effect on the neural response, while the impact of the insertion depth is not observed.

CI global specificity and sensitivity measures were 0.72 ± 0.36 and 0.74 ± 0.35 for the PCM approach, and 0.72 ± 0.04 and 0.75 ± 0.08 for MC. Similarly to the population study, Tables 1, 2 show worst results on the basal and medial electrodes, in terms of local performance and cross-turn stimulation. Both scores showed similar patterns to the ones found in the population study (Figures 8A,B). Despite the higher standard deviation obtained when using PCM, mean values did not differ more than 3 %, providing an acceptable approximation of the mean behavior. Although the MC approach showed less variance, the computational time reached 1,100 h, while PCM took 96 % less (36 h). The use of higher order polynomials was also evaluated. Results from second to sixth order polynomials – from 9 to 49 samples, respectively – obtained specificity values that differed <1%. Mean values obtained were 0.723, 0.724, 0.724, 0.725, 0.719, 0.720, from 2 to 6 order polynomial, while the mean value using MC was 0.727. Local score values differed depending on their position on the array, however overall differences were <5.5%, being the minimum equal to 0.01%. The required computational time increased exponentially: from 15 to 218 h for first and sixth order, respectively.

**Table 1:** Local performance score.

	**Mean PCM**	**Mean MC**	**SD PCM**	**SD MC**
E12	81.0	80.6	37.8	2.9
E11	68.5	68.3	32.0	1.7
E10	76.3	75.7	35.7	4.6
E9	83.8	82.6	8.8	4.6
E8	86.0	85.6	40.2	3.3
E7	83.0	83.0	40.5	4.3
E6	72.3	71.6	35.8	3.8
E5	61.7	63.3	30.0	11.6
E4	87.8	90.2	42.9	6.8
E3	55.3	53.1	25.4	5.8
E2	49.2	49.6	23.1	1.1
E1	58.8	58.0	26.3	4.0

**Table 2:** Cross-turn stimulation score.

	**Mean PCM**	**Mean MC**	**SD PCM**	**SD MC**
E12	92.6	92.4	44.1	2.6
E11	97.8	95.9	45.7	6.1
E10	91.8	90.7	44.0	5.1
E9	85.2	86.5	40.5	3.1
E8	75.3	76.6	36.4	5.0
E7	70.2	71.1	35.0	6.7
E6	82.7	83.4	39.8	4.2
E5	78.8	78.9	37.5	4.1
E4	74.6	77.2	35.4	4.1
E3	82.3	83.3	39.9	3.4
E2	80.0	79.4	37.7	3.8
E1	95.4	95.4	45.4	1.5

Results showed that mean T-levels were approximated with values 240 ± 59 μA and 251 ± 32 μA computed by PCM and MC, respectively. Both approaches presented similar trends regarding each electrode's T-level: lower threshold at the apex (E1–E4) and higher at the first turn (E8–E11). Threshold mean values differed at most 55 μA, in the worst case (E4), while the best approximation was <5 μA (E1, E2, E3, E12). Likewise, C-levels presented lower values at the apex of the cochlea, while highest values were obtained at the medial part.

Mean C-level was 355 ± 71 μA for the PCM approach, in concordance with the behavior observed in Figure 8B, where in order to avoid cross-turn stimulation at the apex and medial part, lower amplitudes are required. This post-implantation level could not be computed for the MC approach, due to the unfeasible required computational time. Post-implantation stimulus levels—mean values—for a patient-specific case are shown in Table 3. Mean values for the C-level stimulus were evaluated in an average patient (mean cochlear shape, insertion and bone resistivity), obtaining global performance measures of 0.80 and 0.72 for sensitivity and specificity, respectively.

**Table 3:** Post-implantation stimulus levels for a patient-specific case using PCM.

	**E1**	**E2**	**E3**	**E4**	**E5**	**E6**	**E7**	**E8**	**E9**	**E10**	**E11**	**E12**
T-level (μA)	221	185	123	226	234	192	272	298	326	317	265	221
C-level (μA)	268	266	302	301	310	372	410	420	460	420	336	284

## 4. Discussion and conclusions

This work aimed at the assessment of parameter variability and uncertainty using a computational framework for the modeling and the evaluation of CI. To this end, we employed uncertainty quantification methods and the developed automatic framework to functionally evaluate the implant in terms of neural excitation. We used a HTC environment to reduce the computational effort of the uncertainty study while evaluating the range of variability on the population.

Initial results showed that 53% of the virtual population obtained global performance measures in terms of specificity within the range [0.70, 0.80], and almost 10% above 0.80. This performance was related to a low rate of false positives, highly desirable in order to avoid confusing pitch for the patients.

Specificity values below 0.5 were related to wider spread of excitation and ANF recruitment due to an increase of bone resistivity, which combined with small cochlear dimensions, caused a considerable amount of non-selective stimulation. This is in line with the findings presented by Tang et al. (2012) and Malherbe et al. (2015). Indeed, results showed the large impact of the bone resistivity over the neural response: as it increases, CI outcomes worsen (i.e., lower performance measure, higher cross-turn stimulation and broader excited pitch). This behavior can be explained by the tendency of the currents to leak from the cochlear structure when the surrounding bone presents a low resistivity value. In those cases, a reduction of the current density and a narrower spread of excitation are observed (Malherbe et al., 2015). As the current leaks, higher post-implantation stimulus levels are required to reach the desired excited pitch (Frijns et al., 2009). In agreement with the findings reported by Tang et al. (2012) and Malherbe et al. (2015), our results showed that consequently, for high resistivity values (absence of bone conduction) lower stimulus intensity should be employed.

Morphology of the cochlea has also shown an impact over the neural response, as suggested by (van der Marel et al., 2014). The first modes of variation of the SSM can be roughly related to the morphology of the inner ear: the variation in general size, the dimension of the spiral radius and the rotation of the cochlea over the rest of the inner ear (the vestibular canals), for the first, second and third mode, respectively (see Figure 2). The second mode is the most influential to the CI outcomes. When it increases, the electrodes are further from the ANF (basal part distances from the modiolus), obtaining a more selective ANF recruitment and better performance measures (Figure 6).

The surgical length of insertion has always been a controversial aspect of the CI procedure. In the clinical practice a high variability of insertion depth has been reported (Gstoettner et al., 2004; Rebscher et al., 2008; Franke-Trieger et al., 2014; Kalkman et al., 2014; van der Marel et al., 2014), which varies according to the implant design, target intra-cochlear position (closer to the modioulus or the lateral wall) and target frequencies (shorter EAs focus on high frequencies, while longer ones cover the whole frequency range). Despite the wide range of reported results, some authors found no significant influence on the patient speech perception (Van Der Marel et al., 2015), while others remarked the insertion depth as a key factor, since it directly affects the alignment between frequency and cochlear location (Dorman et al., 1997; Finley et al., 2008; Mangado et al., 2017b). We found that the impact of the insertion depth was subtle, and mainly observed at the base of the cochlea. This was caused by the narrow spread of excitation, which missed some target frequencies.

Although the computational quantification of the implant performance has not been attempted before, local effects have been previously reported. As suggested by Frijns et al. (2001) and Briaire and Frijns (2006), we observed that electrode contacts in the last cochlear turn presented cross-turn stimulation at the base of the cochlea – caused by the tightly coiled geometry of the cochlea at the apex. In addition, medial and basal electrodes showed cross-turn stimulation, identified to be related to the excitation of lower pitches. This could be explained by the use of a high impulse intensity, which combined with the low bone conduction, generates wider current fields that excite a high amount of non-selective ANF. Indeed, we observed that a wider excitation area tends to appear at the apex, as indicated by van der Beek et al. (2012) and Biesheuvel et al. (2016), which limits the spatial selectivity at the apex (Briaire and Frijns, 2006). Results agreed with reported excited pitches for similar computational conditions: lateral electrodes produced similar excitation pitch for bandwidths of 4 mm, i.e., E7 and E10 generated a pitch of 800–1,500 Hz and 2,100–4,400 Hz, respectively, in concordance with 900–1,700 Hz, and 2,000–4,000 Hz reported by Kalkman et al. (2014). These variations could be explained by a slight difference of the angular insertion depth. However, frequency bandwidth wider than 3 mm should be avoided since it implies a change of one octave in frequency, causing a high confusing pitch and therefore a large impact in CI outcomes (Mistrík and Jolly, 2016). To avoid this, in the clinical practice optimal stimulus amplitudes are sought to reach the desired pitch at each electrode location.

Results showed that lower amplitudes were required at apical electrodes, in line with Brill et al. (2009), Malherbe et al. (2013), Kalkman et al. (2014), and van der Beek et al. (2016). Predicted levels tended to decrease on the first electrodes, while increasing toward the base (Malherbe et al., 2013; van der Beek et al., 2016). Obtained T-levels can be compared with experimental measurements (eCAP thresholds): from 190 μA at the apex to 460 μA at the base for a Med-EL Flex28 array (Brill et al., 2009). These findings are also in agreement with previous computational studies, which found T-levels from 150 to 400 μA (Kalkman et al., 2014). However, they also reported relevant differences on these levels according to the geometrical description of the ANF, defined either as radial or oblique trajectories (Kalkman et al., 2014, 2015). The latter provided a better representation of the ANF by relating more accurately the peripheral process of each ANF with the position of its cell body in the spiral ganglion (Stakhovskaya et al., 2007; Kalkman et al., 2015). We believe that the improvement of such trajectories could explain some discrepancies of our results with the clinical data. In addition, previous studies defined the T-level and C-level as the stimulus required to evoke a bandwidth of 1 and 4 mm along the basilar membrane, respectively (Briaire and Frijns, 2006; Kalkman et al., 2014), based on experimental findings reported by Snel-Bongers et al. (2013). Although our proposed performance measures penalized the occurrence of cross-turn stimulation, including this information into our description could provide more reliable post-implantation levels.

The developed framework has a high cost, specifically when a large set of samples needs to be evaluated. The parallelization of the framework to conduct the population study using a HTC environment allowed processing all data more efficiently (4.9 times faster). Still, there is room for improvement. While providing a detailed description of the neural behavior in CI models, the implemented ANF model implied a high computational effort (Hanekom and Hanekom, 2016). Less-expensive neural models, such as analytical or single-compartment models, could provide an alternative to reduce the required time of simulation. Although these models have been also used for the generation of the action potential (Brette, 2015), they are less realistic and they could imply some limitations on the CI assessment in patient-specific studies, such as in cases of ANF degeneration (Rattay et al., 2001a,b).

As for the uncertainty propagation approach, other sampling techniques could be used instead of MC to reduce the number of runs needed and, therefore, the overall required computational time (Berthaume et al., 2012). The appropriate number of samples to evaluate depends on each case study (Sarrazin et al., 2017), fact that makes it difficult to ensure the desired accuracy without conducting a prior dimensional analysis. The computational effort of the implemented framework hampers such analysis. However, our results are in line with previous findings, thus we consider the set of 250 samples evaluated an acceptable approximation.

Whereas PCM provided a trade-off between computational time and precision in the patient-specific case –compared to the mean obtained by the MC sampling approach–, the population study involved more uncertainty sources, which implied an exponential increment on the computational time. For this reason, PCM is recommended only for studies with few uncertain parameters, since otherwise the benefit of using a considerably lower number of runs than MC would be reduced. Results using PCM had a larger standard deviation. Polynomials of order higher than 6 should be used to gain in accuracy. However, the required computational time increases exponentially, and therefore, the advantage of using PCM to obtain the mean response of the system would be drastically reduced.

Additionally, other approaches for the uncertainty analysis can be employed, for instance, intrusive methods, which reformulate and solve the stochastic version of the deterministic FE model (Mangado et al., 2016b). They have been implemented successfully in electrical simulations considering sources of uncertainty the tissue electrical properties (Geneser et al., 2008) or the behavior of the ionic channels that control cardiac contractions (Du and Du, 2016). Despite their limitation when considering geometrical aspects, they may provide faster solutions to assess patient-specific cases.

Although implant performance in CI has been rarely quantified computationally due to the several involved physiological effects, results suggest that the proposed framework provides reliable information regarding the behavior of the implanted cochlea and in concordance with previous computational and experimental findings. Further improvements include the use of trains of pulses as electrical stimulus inducing then a temporal neural response, as well as the evaluation of different stimulation protocols in terms of current focusing and selective neural recruitment. This study has analyzed the influence of EA insertion and bone resistivity uncertainty according to the variation of the cochlear morphology among the population. This information can help surgeons to select the surgical parameters to achieve the optimal outcome of CI (Finley et al., 2008; van der Marel et al., 2014). Moreover, this work may provide a powerful computational tool for implant design optimization purposes, as well as for the implant programming to establish the most suitable stimulation setting. Overcoming the limitations mentioned above would lead to a more precise and highly accurate computational tool for its use in the clinical practice.

## Author contributions

NM, JP-P, MCe, and MG: contributed conception and design of the study; NM, JP-P, and MCo: developed the uncertainty quantification study and its implementation in the HTC environment; NM: performed the computational studies, processed the data and obtained results; NM, MCe, PM, and MG: interpreted the results and discussed the resulting conclusions; NM: wrote the first draft of the manuscript and all authors contributed to the manuscript revision, read and approved the submitted version.

### Conflict of interest statement

Author PM was employed by company MED-EL, Austria. The other authors declare that the research was conducted in the absence of any commercial or financial relationships that could be construed as a potential conflict of interest.
